# Local Phase Segregation Induced by Ion Milling in 2:17-Type Sm-Co Based Magnets

**DOI:** 10.3390/ma16124378

**Published:** 2023-06-14

**Authors:** Xin Song, Yao Liu, Wentao Jia, Jian Li, Xiaolian Liu, Lizhong Zhao, Tao Yuan, Tianyu Ma

**Affiliations:** 1 Frontier Institute of Science and Technology and State Key Laboratory for Mechanical Behavior of Materials, Xi’an Jiaotong University, Xi’an 710049, China; 2 College of Materials and Environmental Engineering, Hangzhou Dianzi University, Hangzhou 310018, China; 3 The Southwest Applied Magnetism Research Institute of China, Mianyang 621000, China

**Keywords:** Sm-Co magnets, ion milling, phase segregation, TEM

## Abstract

Transmission electron microscopy (TEM) is indispensable to reveal the cellular nanostructure of the 2:17-type Sm-Co based magnets which act as the first choice for high-temperature magnet-associated devices. However, structural deficiencies could be introduced into the TEM specimen during the ion milling process, which would provide misleading information to understand the microstructure–property relationship of such magnets. In this work, we performed a comparative investigation of the microstructure and microchemistry between two TEM specimens prepared under different ion milling conditions in a model commercial magnet Sm_13_Gd_12_Co_50_Cu_8.5_Fe_13_Zr_3.5_ (wt.%). It is found that additional low-energy ion milling will preferably damage the 1:5H cell boundaries, while having no influence on the 2:17R cell phase. The structure of cell boundary transforms from hexagonal into face-centered-cubic. In addition, the elemental distribution within the damaged cell boundaries becomes discontinuous, segregating into Sm/Gd-rich and Fe/Co/Cu-rich portions. Our study suggested that in order to reveal the true microstructure of the Sm-Co based magnets, the TEM specimen should be carefully prepared to avoid structural damage and artificial deficiencies.

## 1. Introduction

Due to the excellent magnetic performance at elevated temperature and good correction resistance, the 2:17-type Sm-Co based magnets have been the first choice for high-temperature magnet-associated applications, such as traveling wave tubes (TWT) for space exploration and satellite communication, inertial devices for accelerometers and gyroscopes, and permanent magnet motors [1,2,3,4,5,6]. The fabrication of the 2:17-type Sm-Co magnets involves conventional powder metallurgy, including induction melting, crushing, and compressing in a magnetic field, followed by a complex heat treatment process, containing sintering, solution treatment, aging, and slow cooling. Then, a unique cellular nanostructure will be developed in the final magnets, which has been widely deemed as the origin of the hard magnetic properties for the 2:17-type Sm-Co based magnets which are closely related to unique cellular nanostructure, consisting of the Fe/Co-rich 2:17R (Th_2_Zn_17_ structure, R$\bar{3}$m) cell interior, Sm/Cu-rich 1:5H (CaCu_5_ structure, P6/mmm) cell boundary phase, and intercrossing Zr-rich 1:3R (NbBe_3_ structure, R$\bar{3}$m) planar Z-phase [4,5,7,8,9]. The coercivity mainly arises from the domain wall energy density between the 1:5H phase and 2:17R phase [7,8,9]. In addition, the nano-sized structural defects generated during the phase decomposition process also play an important role in the magnetic properties of the 2:17-type Sm-Co based magnets, especially the remanent stacking faults at the cell edge [10,11,12]. Since these crystalline phases and defects are very small in size (e.g., the thickness of the 1:5H phase is usually smaller than 10 nm) and the structures of the coherent 1:5H and 2:17R phases are similar and their lattice parameters are approximately equal [13,14], it is difficult to perform the specific microstructure by scanning electron microscopy or conventional X-ray diffraction technologies. Thus, transmission electron microscopy (TEM) and high-resolution transmission electron microscopy (HR-TEM) are indispensable to reveal the true structure of the 2:17-type Sm-Co based magnets [3,4,5,7,8,9,10,11,12,13,15].

However, the TEM (or HR-TEM) is not risk-free, in terms of introducing microstructural artifacts, especially during the TEM specimen preparation process. Generally, the thin film specimens for TEM examinations of the 2:17-type Sm-Co based magnets are fabricated by ion milling or focused ion beam, since these magnets are too brittle to suffer electropolishing [4,5,7,8,10,11,12,13]. Although the energy used for ion milling or focused ion beam is relatively low (2–6 keV), the possible structure damage can be easily introduced into metallic materials since the temperature rising of the specimen surface is unavoidable during the ion milling process. In addition, many studies in ion implantation/ion beam modification of materials point out that the structure of the specimens might become modified by energetic ions, and artifacts can be formed in this way [16,17,18,19,20,21]. Besides the widely known amorphous layer near the hole, there are other artifacts including defects clusters [17], elemental segregation [18], and phase transformation [19] that can be observed during the particle irradiation process. Note that the high-performance magnets will suffer damaging environmental effects, especially in applications such as space probes, high energy particle accelerators, spectrometers, and new synchrotron light sources; thus, the potential structure damage induced by ion irradiation must be considered. Previous investigations about the Sm-Co based magnets all focus on the high energy heavy ion irradiation damage, indicating that the magnetic properties, especially the magnetization, drop drastically when these magnets suffer from high energy irradiation [3,22,23]. However, the possible structure damage induced by low-energy ion irradiation in the 2:17-type Sm-Co based magnets are rarely considered.

Herein, we performed a comparative investigation between two TEM specimens fabricated by low-energy ion milling under different conditions of a commercial magnet Sm_13_Gd_12_Co_50_Cu_8.5_Fe_13_Zr_3.5_ (wt.%). The detailed structure and composition of these specimens are unveiled using TEM equipped with an energy dispersive X-ray spectroscopy (EDS) detector. Our study not only suggests that careful parameters of ion milling should be selected during the TEM specimen preparation process, but also provides new understanding of the 2:17-type Sm-Co magnets.

## 2. Materials and Methods

A commercial magnet with a nominal composition of Sm_13_Gd_12_Co_50_Cu_8.5_Fe_13_Zr_3.5_ (wt.%) was provided by the Southwest Applied Magnetism Research Institute of China (Mianyang, China). The ingot was prepared using the conventional powder metallurgy method by induction melting high purity (99.9%) constituent elements under the protection of high purity argon with 5 wt.% more Sm added to compensate for the Sm volatilization. Then, the as-cast ingot was crushed into powders with an average size of ~4–6 μm by jaw crusher and jet-milling. The obtained powders were pressed under ~150 MPa in a magnetic field higher than 10 kOe, followed by isostatic compaction under ~200 MPa. Subsequently, the green compact was sintered for ~1 h at ~1215 °C, and solution-treated for ~4 h at ~1175 °C, followed by cooling to room temperature using high-speed argon flow. Then, the solution-treated magnet was aged for 24 h at ~810 °C and slow cooled to room temperature.

The average structure of the powder magnet was characterized using a Rigaku X-ray diffractometer with Cu K_α_ radiation. Two slices were cut from the final magnet along the [001] axis for TEM specimen preparation. The TEM specimens were prepared by standard mechanical polishing, dimpling to a thickness of 20–30 μm, and followed by ion milling by Ar^+^ in a Gatan 691 precision ion polishing system (PIPS). One specimen (named S1) was milled at 5 keV for each gun with an incident angle of 10° until hole perforation was achieved. The other (named S2) was also milled under the same conditions as specimen S1 before hole perforation, and then milled at 3 keV for each gun with an incident angle of 6° for 15 min. The phase structure and elemental distribution were identified using a JEOL 2100F TEM (JEOL, Tokyo, Japan) and a Thermo Fisher Talos F200S high-resolution scanning TEM (HR-STEM) (Thermo Fisher, Waltham, MA, USA) equipped with an EDS detector. The HR-TEM images were analyzed using digital micrography software (Version 3.22.1461.0, Gatan).

## 3. Results

### 3.1. Average Structure of the Final Magnet

**Figure 1:**
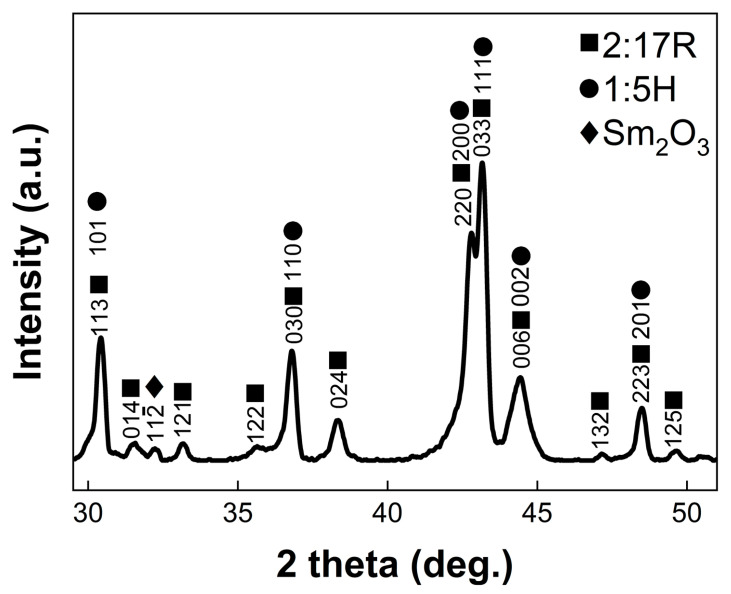
Powder XRD patterns of the final magnet.

As shown in Figure 1, the powder XRD pattern was taken to assess the average structure of the final magnet. All the fundamental reflections of the 2:17R and 1:5H phases are overlapped with each other since these two phases are coherent with each other ([100]-2:17R // [210]-1:5H, (003)-2:17R // (001)-1:5H) and the difference between the lattice parameters of them are small (*a*/$\sqrt{3}$-2:17R ≈ *a*-1:5H, *c*/3-2:17R ≈ *c*-1:5H) [14]. It also exhibits weak superlattice reflections unique to the 2:17R phase, such as the {014}-2:17R at 2 theta of ~31.5° and {024}-2:17R at 2 theta of ~38.4°. Meanwhile, the 1:3R phase is too small (the thickness is usually smaller than 10 nm) to be detected by conventional XRD [24,25]. The {11$\bar{2}$} reflection at 2 theta of ~32.2° belongs to the Sm_2_O_3_ primary precipitates. During the standard preparation process of the 2:17-type Sm-Co based magnets, the Sm will be inevitably oxidated by the trace oxygen, resulting in the formation of the Sm_2_O_3_ primary precipitates [26].

### 3.2. Microstructure and Microchemistry for Both TEM Specimens

**Figure 2:**
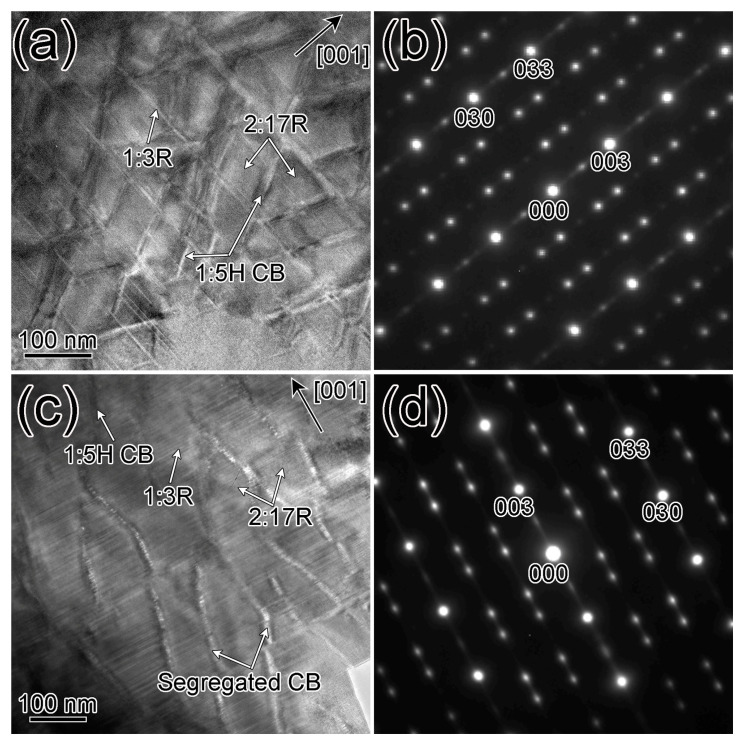
Bright field images, selected area electron diffraction patterns for specimen 1 (**a**,**b**) and specimen 2 (**c**,**d**).

Figure 2 shows the bright-field images (BFIs) and the selected area electron diffraction (SAED) patterns of the two TEM specimens taken along [001]-2:17R zone axis. To perform the real structure to the most extent, two SAED patterns were taken using the largest aperture of our TEM to include diffraction information of an area with a diameter of ~4 μm. In the SAED patterns [Figure 2b,d], the 1:5H phase produces only fundamental reflections ({001}- and {1$\bar{2}$0}-1:5H) which are overlapped by the fundamental reflections of the 2:17R phase ({003}- and {1$\bar{2}$0}-1:5H), which is consistent with the XRD results in Figure 1. The superlattice reflections at {011} *, {012} *, {021} *, and {022} * positions are attributed to the 2:17R nano twins. The 1:3R phase produces extra superlattice reflections {003}-1:3R at the {003/2}*-2:17R positions. The orientation relationship of these three phases is [100]-2:17R // [210]-1:5H/1:3R and (006)-1:3R // (003)-2:17R // (001)-1:5H. The additional weak reflections at the positions of {001} *-, {002} *-, {010} *-, {020} *-, {031} *-, {032} *-, {013} *- and {023} *-2:17R are attributed to the 2:17R’ phase, which is an intermediate during the transformation from the supersaturated solid solutions to the 2:17R equilibrium phase [10,11]. The BFIs (Figure 2a,c) reveal that both specimens have a diamond-shaped cellular nanostructure, consisting of the 2:17R cell interiors, the cell boundaries along the {011}-2:17R pyramidal plane, and the 1:3R platelets along the {001}-2:17R basal planes. The cell boundaries in S1 (Figure 2a) all exhibit dark contrast which can be deemed as the 1:5H phase. However, the cell boundaries near the hole in S2 (right region in Figure 2c) exhibit alternately bright and dark contrasts, which are rarely observed in the 2:17-type Sm-Co based magnets, while the cell boundaries far away from the hole exhibit a dark contrast. The abnormal microstructure in specimen S2 indicates that the structure and chemistry of the cell boundaries near the hole may be altered during the relatively lower energy pips process.

**Figure 3:**
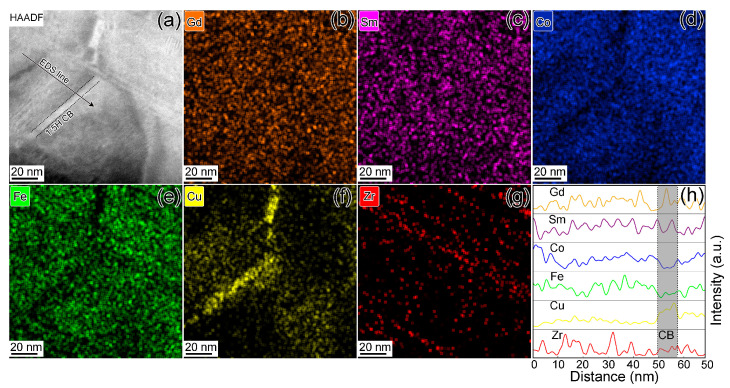
STEM-HAADF image taken along [100]-2:17R zone axis (**a**), the corresponding elemental mapping images (**b**–**g**), and line-scanning EDs results (**h**) along the black arrow in (**a**) for specimen 1.

**Figure 4:**
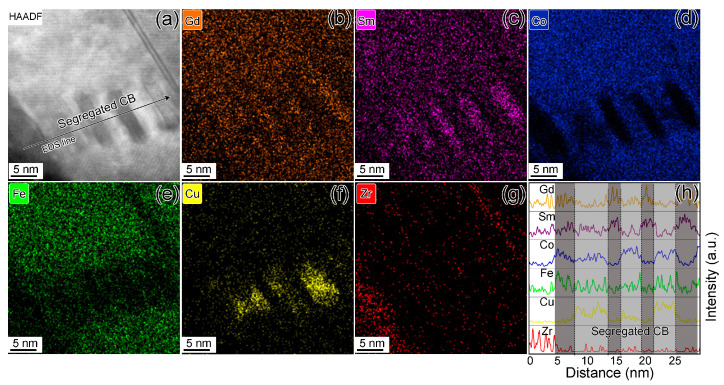
STEM-HAADF image taken along [100]-2:17R zone axis (**a**), the corresponding elemental mapping images (**b**–**g**), and line-scanning EDS results (**h**) along the black arrow in (**a**) for specimen 2.

The bright-field images shown in Figure 2 were selected in an area with a size of ~500 × 500 nm^2^ to show the cellular structure clearly; however, this region is too large to perform the microchemistry near the cell boundaries region. Thus, higher-resolution scanning TEM high-angle annular dark field (STEM-HAADF) images and the corresponding elemental distribution for both specimens were performed in Figure 3 and Figure 4, respectively. The cell boundaries are shown in in Figure 3a; the bright contrasts along the {011} pyramidal planes can be deemed as 1:5H phases. The corresponding elemental mapping images in Figure 3b–g further reveal that Cu is distinctly concentrated at the cell boundary phase with relatively depleted Fe and Co at the cell boundaries in S1, while the cell interior is Fe/Co-rich and Co-depleted. The Zr-rich 1:3R platelets along the {001} basal planes may act as the elemental distribution paths to form the chemistry heterogeneous between the cell interiors and cell boundaries [4,27,28]. The TEM-EDS line scanning along the black arrow in Figure 3h shows the elemental distribution difference more clearly. The marked region (gray rectangle region in Figure 3h) refers to the cell boundary, in which the Cu concentration is higher and Fe/Co concentration is lower than those in the cell interior.

Figure 4a shows the STEM-HAADF image of specimen S2. It can be seen that the cell boundaries in S2 are distinctly different from those in S1. The cell boundaries are segregated into two types of lamellae (with a thickness of ~2–5 nm and a length of ~10 nm) with bright and dark contrasts. It is well known that the brightness of STEM-HAADF images is proportional to Z^1.6–1.9^ (Z being the atomic number) [29]. Consequently, the bright contrasts are assembled with higher Z Gd and Sm atoms, while the dark contrasts are assembled with lower Z Co, Fe, and Cu atoms. As confirmed from the elemental mapping images Figure 4b–g, the segregated cell boundaries are composed of Gd/Sm-enriched and Co/Cu/Fe-enriched lamellae, and the elemental distribution among the cell interiors seems homogeneous. Figure 4h performs the line scanning elemental distribution across the segregated cell boundary along the arrow in Figure 4a. The gray regions with different contrast represent the segregated cell boundaries with different element concentrations. Some of the cell boundaries are enriched in Gd and Sm elements but lacking in Fe, Co, and Cu elements, while the elemental distribution in the other cell boundaries is inverse. The segregated elemental distribution indicates that the atom diffusion is affected by the ion irradiation during the low-energy ion milling process.

### 3.3. Atomic-Scale Structure Identification of the CBs in Both TEM Specimens

**Figure 5:**
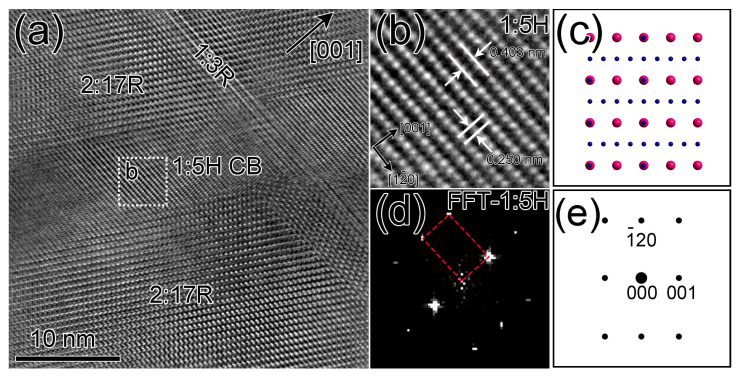
HR-TEM image near the cell boundary region for the specimen 1 (**a**), enlarged view (**b**) of the white dashed square region in (**a**), and FFT pattern (**d**) taken from the dashed square region in (**a**). [210]-SmCo_5_ projection (**c**) and simulated electron diffraction pattern (**e**) for SmCo_5_ phase.

**Figure 6:**
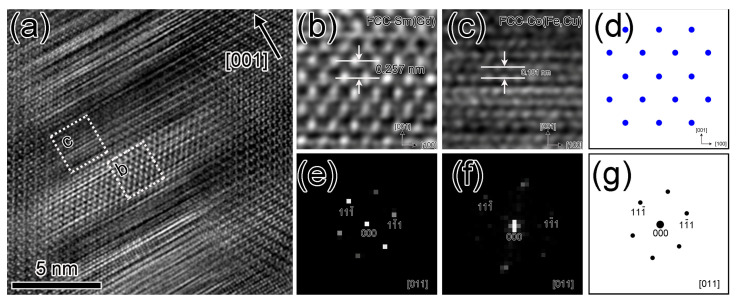
HR-TEM image near the cell boundary region for the specimen 2 (**a**), enlarged views (**b**,**c**) of the white dashed square regions in (**a**), and FFT patterns (**e**,**f**) taken from dashed square regions in (**a**). [011]-FCC structure projection (**d**) and simulated electron diffraction pattern (**g**) for FCC structure.

To further identify the structure of different cell boundaries, HR-TEM characterizations for both TEM specimens were performed, as shown in Figure 5 and Figure 6. As shown in the HR-TEM image of the cell boundary region of S1 (Figure 5a), a homogeneous and continuous cell boundary is observed along the {011} pyramidal plane, having a ~60° angle to the {001} basal plane. The corresponding enlarged view and fast Fourier transformation (FFT) pattern taken from the white dashed square region in Figure 5a is performed to justify the atomic structure. The enlarged view shows that the Sm atoms with bright contrasts have assembled the 1:5H lattice, which is consistent with the [210]-1:5H projection shown in Figure 5c. The FFT pattern indexed by red dashed lines in Figure 5d contains only fundamental reflections, which is also consistent with the simulated electron diffraction pattern for SmCo_5_ phase (Figure 5e). The measured lattice spacings based on the HR-TEM image are *d* (001) ≈ 0.403 nm and *d* (1$\bar{2}$0) ≈ 0.250 nm. Then, the calculated lattice parameters are *a* ≈ 0.500 nm and *c* ≈ 0.403 nm. The difference between the calculated lattice parameters and the lattice parameter of binary SmCo_5_ can be attributed to the combined effect of the substitution of Gd to Sm and the substitution of Cu to Co. The above analysis in both reciprocal and real spaces demonstrates that the cell boundaries in the TEM specimen S1 are the 1:5H phase.

In contrast, the detailed atomic-scale structure of the cell boundary in the TEM specimen S2 is performed in Figure 6. The segregated cell boundaries in Figure 6a also occupy the {011} pyramidal plane. The structure of the 2:17R cell interiors phase is unchanged. The enlarged views of these two lamellar structures and corresponding FFT patterns are shown in Figure 6b,c,e,f, respectively. Both structures exhibit an FCC structure judged by atom stacking sequence in real space and FFT pattern in reciprocal space. According to the above STEM-HAADF images and elemental mapping images (Figure 3 and Figure 4), the lamella with bright contrast is enriched with Gd and Sm elements, while the other with dark contrast is enriched with Co, Fe, and Cu elements. Consequently, the former can be deemed as an FCC-Sm(Gd) structure, whose lattice parameter is ~0.514 nm (calculated from the measured lattice spacing value in Figure 6b, *d* (001)/2 ≈ 0.257 nm), and this value is smaller than that for the FCC-Gd structure (0.532 nm) and slightly larger than that for the FCC-Sm structure (0.512 nm) [30]. The latter can also be identified as an FCC-Co (Fe, Cu) structure with a lattice parameter of 0.362 nm, which is also nearly equal to that for the γ-Fe (0.361 nm) [31], FCC-Co (0.355 nm) [32], or FCC-Cu (0.362 nm) [33] structures.

## 4. Discussion

The above comparative investigations between two TEM specimens with different ion milling conditions but the same composition and same heat treatment history reveal that the primary 1:5H cell boundaries near the hole in the TEM specimen with additional low-energy ion milling will transform from hexagonal into face-centered-cubic. In addition, the elemental distribution within the damaged cell boundaries becomes discontinuous, segregating into Sm/Gd-rich and Fe/Co/Cu-rich portions. The possible phase segregation mechanism is discussed below.

The formation of the cellular nanostructure in the 2:17-type Sm-Co magnets was deemed as a concurrent recrystallization and precipitation process, which involved the growth of the 2:17R cell interior phase (sub-grain interior) and the precipitate of the 1:5H phase along the cell boundaries (sub-grain boundaries) [5,8,9]. The whole process is controlled by a coupled diffusive and displacive phase transformation. The motion of the partial dislocations transforms the 2:17H solution-treated precursor into the 2:17R phase, leaving a number of interstitials and vacancies in the {011} pyramidal planes, which act as the nucleation site for the 1:5H precipitates. In addition, the elemental partition occurs, including the rare earth atoms (Sm or Gd) gathering into the pyramidal planes, the Fe and Co atom enrichment into the cell interiors, and the Zr atoms gathering into the {001} basal planes to form the 1:3R platelets. During the ion milling process, the temperature-rising effect contained with the bombardment of the argon ion may offer extra driving force to the atom’s diffusion, which can change the structure and microchemistry of the 1:5H cell boundaries phase. Although the energy for the TEM specimen’s preparation is too low to induce structural artifacts into the 2:17-type Sm-Co based magnets since the irradiation resistance of these magnets is good enough at room temperature, the temperature-rising effect during the ion milling cannot be ignored. Earlier investigation [34] implied that elevated temperature (200–400 °C) can be easily generated at the specimen surface under a normal ion milling process, even for the metallic specimens with good thermal conductors. In addition, the thermal effect is induced by the incoming ion bombardments to the specimen surface [35]. Consequently, the possible ion damage at high temperatures must be considered for the Sm-Co based magnets during the low-energy ion milling process. According to previous investigation, the formation of the 1:5H phase is highly controlled by the diffusion of the interstitial atoms [8]. On the one hand, the rising temperature during the ion milling (especially after the hole perforation is achieved for specimen 2) may provide extra freedom to alter the atom diffusion process; e.g., the extra temperature field enlarges the thermodynamic driving force. On the other hand, the mechanical interaction between the Ar^+^ and Sm/Gd/Cu/Fe/Co atoms in the hole-edge region with an extremely small thickness also may change the diffusion behavior. It should be noted here that ion milling of specimens for TEM examination is not a static process, since the outer surfaces of the specimen are continually being eroded by the ion beam. Thus, these combined effects in the hole-edge region in specimen 1 induce further atom diffusion, resulting in cell boundaries segregation from the 1:5H phase to the Sm/Gd-rich and Co/Fe/Cu-rich FCC structures. Since the ion milling energy used in this work was determined empirically, the systematic effect of the ion milling energy on the structure and microchemistry of the TEM specimen needs to be further investigated.

This work explored the structural damage that occurs during ion milling in the preparation of the TEM specimens for 2:17-type Sm-Co based magnets, rarely done by previous investigations, which may also provide new insight for the widely-investigated irradiation-induced demagnetization for these magnets [3,22,23]. This work also emphasizes the importance of careful control for the ion milling conditions and the design of advanced ion milling strategy to avoid erroneous interpretation in TEM-related studies. 

## 5. Conclusions

The low-energy ion milling-induced local phase segregation in the preparation of the TEM specimen in a model Sm_13_Gd_12_Co_50_Cu_8.5_Fe_13_Zr_3.5_ (wt.%) magnet was revealed. Based on the detailed comparative investigations, it can be concluded that the 1:5H cell boundaries near the hole of the TEM specimen will segregate into an Sm/Gd-rich FCC structure and a Fe/Co/Cu-rich FCC structure during the additional low-energy ion milling process. This finding suggests that artifacts should be carefully avoided to identify the cellular nanostructure of 2:17-type Sm-Co based magnets. In addition, our investigation also revealed that the 1:5H cell boundary phase is easier to damage when compared with the 2:17R phase, despite their structural similarities, which may also be helpful to evaluate the magnet operation performance in irradiation environments.

## Data Availability

Data sharing is not applicable.

## References

[1] Ojima T., Tomizawa S., Yoneyama T., Hori T. (1977). Magnetic properties of a new type of rare-earth cobalt magnets Sm_2_(Co, Cu, Fe, M)_17_. IEEE Trans. Magn..

[2] Gutfleisch O., Willard M.A., Brück E., Chen C.H., Sankar S., Liu J.P. (2011). Magnetic materials and devices for the 21st century: Stronger, lighter, and more energy efficient. Adv. Mater..

[3] Gao R.S., Zhen L., Shao W.Z., Hao X.P., Sun X.Y., Yang L., Wang B.Y. (2008). Study of γ-ray irradiation effect on permanent magnets. J. Appl. Phys..

[4] Xiong X.Y., Ohkubo T., Koyama T., Ohashi K., Tawara Y., Hono K. (2004). The microstructure of sintered Sm(Co_0.72_Fe_0.20_Cu_0.055_Zr_0.025_)_7.5_ permanent magnet studied by atom probe. Acta Mater..

[5] Sepehri-Amin H., Thielsch J., Fischbacher J., Ohkubo T., Schrefl T., Gutfleisch O., Hono K. (2017). Correlation of microchemistry of cell boundary phase and interface structure to the coercivity of Sm(Co_0.784_Fe_0.100_Cu_0.088_Zr_0.028_)_7.19_ sintered magnets. Acta Mater..

[6] Livingston J.D., Martin D.L. (1977). Microstructure of aged (Co, Cu, Fe)7 Sm magnets. J. Appl. Phys..

[7] Duerrschnabel M., Yi M., Uestuener K., Liesegang M., Katter M., Kleebe H.J., Xu B., Gutfleisch O., Molina-Luna L. (2017). Atomic structure and domain wall pinning in samarium-cobalt-based permanent magnets. Nat. Commun..

[8] Song X., Ma T.Y., Zhou X.L., Ye F., Yuan T., Wang J.D., Yue M., Liu F., Ren X.B. (2021). Atomic scale understanding of the defects process in concurrent recrystallization and precipitation of Sm-Co-Fe-Cu-Zr alloys. Acta Mater..

[9] Gopalan R., Hono K., Yan A., Gutfleisch O. (2009). Direct evidence for Cu concentration variation and its correlation to coercivity in Sm(Co_0.74_Fe_0.1_Cu_0.12_Zr_.04_)_7.4_ ribbons. Scr. Mater..

[10] Jia W.T., Zhou X.L., Xiao A.D., Song X., Yuan T., Ma T.Y. (2020). Defects-aggregated cell boundaries induced domain wall curvature change in Fe-rich Sm-Co-Fe-Cu-Zr permanent magnets. J. Mater. Sci..

[11] Xu C., Wang H., Liu B.J., Xu H., Zhang T.L., Liu J.H., Jiang C.B. (2020). The formation mechanism of 1:5H phase in Sm(Co, Fe, Cu, Zr)_z_ melt-spun ribbons with high iron content. J. Magn. Magn. Mater..

[12] Li Q.F., Wang C., Lin Z.C., Wang L., Xiao Y.F., Zhang P., Cao X.Z., Fang Y.K., Liao Q.L., Yang J.B. (2023). Phase separation and chemistry evolution in Fe-rich Sm(Co_bal_Fe_0.31_Cu_0.07_Zr_0.025_)_7.7_ magnets: The effect of initial defect. Acta Mater..

[13] Song X., Huang D., Jia W.T., Liu Y., Gao J.R., Ren Y., Ma T.Y. (2023). In-situ high-energy X-ray diffraction study of the early-stage decomposition in 2:17-type Sm-Co-based permanent magnets. Acta Mater..

[14] Buschow K.H.J., Van der GOOT A.S. (1968). Intermetallic compounds in the system samarium-cobalt. J. Less Common Met..

[15] Zhang W., Chen H.Y., Song X., Ma T.Y. (2021). Grain boundary evolution of cellular nanostructured Sm-Co permanent magnets. Acta Mater..

[16] Barber D.J. (1993). Radiation damage in ion-milled specimens: Characteristics, effects and methods of damage limitation. Ultramicroscopy.

[17] Robertson I.M., Vetrano J.S., Kirk M.A., Jenkins M.L. (1991). On the formation of vacancy type dislocation loops from displacement cascades in nickel. Philos. Mag. A.

[18] Van Wyk G.N., Du Plessis J., Taglauer E. (1991). Sputtering and radiation-enhanced segregation in amorphous Cu-Ti alloys. Surf. Sci..

[19] Belkacemi L.T., Meslin E., Décamps B., Radiguet B., Henry J. (2018). Radiation-induced bcc-fcc phase transformation in a Fe-3%Ni alloy. Acta Mater..

[20] Sun B.B., Wang Y.B., Wen J., Yang H., Sui M.L., Wang J.Q., Ma E. (2005). Artifacts induced in metallic glasses during TEM sample preparation. Scr. Mater..

[21] Baba-Kishi K.Z., Tai C.W. (2005). Radiation damage by Ar^+^ ion-milling in ferroelectric oxides. Microsc. Microanal..

[22] Zhao X., Li J.Y., Wang L., Sun X.Y., Yang L., Shao W.Z., Yuan T., Xu C.Y., Zhen L. (2023). Effects of neutron irradiation and high temperature on structure and magnetic properties of Sm2Co17 permanent magnet. Nucl. Instrum. Methods Phys. Res. B.

[23] Kähkönen O.P., Kautto E., Manninen M., Talvitie M. (1992). Effects of high temperature irradiation on SmCo permanent magnets. J. Appl. Phys..

[24] Maury C., Rabenberg L., Allibert C.H. (1993). Genesis of the cell microstructure in the Sm(Co, Fe, Cu, Zr) permanent magnets with 2:17 type. Phys. Status Solidi A.

[25] Kronmüller H., Goll D. (2002). Micromagnetic analysis of pinning-hardened nanostructured, nanocrystalline Sm_2_Co_17_ based alloys. Scr. Mater..

[26] Zhang Y., Wu P.F., Ming W.Q., Cao X., Huang Y.Z., Li Z.M. (2023). On the structure of rare-earth sesquioxide Sm_2_O_3_ in Sm2Co17-type magnets. Scr. Mater..

[27] De Campos M.F., Murakami R.K., Romero S.A., Rechenberg H.R., Missell F.P. (2007). Magnetic characterization of the (Zr,Sm)Co_3_ phase in Sm(CoFeCuZr)_z_ magnets. J. Appl. Phys..

[28] Tang W., Zhang Y., Hadjipanayis G.C. (1999). Effect of Zr on the microstructure and magnetic properties of Sm(Co_bal_Fe_0.1_Cu_0.088_Zr_x_)_8.5_ magnets. J. Appl. Phys..

[29] Nellist P.D., Pennycook S.J. (1999). Incoherent imaging using dynamically scattered coherent electrons. Ultramicroscopy.

[30] Kirklin S., Saal J.E., Meredig B., Thompson A., Doak J.W., Aykol M., Rühl S., Wolverton C. (2015). The Open Quantum Materials Database (OQMD): Assessing the accuracy of DFT formation energies. npj Comput. Mater..

[31] Schlosser W.F. (1973). Calculation of the Atomic Volumes of Fe-Ni and Fe-Co Alloys. Phys. Status Solidi A.

[32] Gebhardt E., Köster W. (1940). Das System Platin-Kobalt mit besonderer Berücksichtigung der Phase CoPt. Z. Met..

[33] Luo H.L., Duwez P.E. (1964). Solid solutions of rhodium with copper and nickel. J. Less Common Met..

[34] Kim M.J., Carpenter R.W. (1987). Tem specimen heating during ion beam thinning: Microstructural instability. Ultramicroscopy.

[35] Wang C.Z., Smith D.J. (2006). Understanding ion-milling damage in Hg_1−x_Cd_x_Te epilayers. J. Appl. Phys..

